# A Treatise on the Diseases of the Eye

**Published:** 1833-10

**Authors:** 


### MEDICAL QUARTERLY REVIEW.

### REVIEWS.

*A Treatise on the Diseases of the Eye.* By W. LAWRENCE, F.R.S., Surgeon to St. Bartholomew's Hospital, &c. London, 1833. 8vo. pp. 730.

SEVERAL years have now elapsed since Mr. Lawrence's Lectures on the Diseases of the Eye were published in the Lancet, and we are glad to find that this eminent practitioner has devoted a part of the intervening time to the indispensable task of polishing and correcting.

Mr. Lawrence observes too, "that the opinions and experience of others are quoted and examined;" but it may be fairly objected that he has consulted books too much, and that consequently, in many parts of the treatise, we seem rather to be reading a dictionary of opinions, than the spontaneous effusions of a master of the art. Homogeneousness (the characteristic of genius,) is the great charm of Beer's work. Not that we are to suppose this distinguished teacher to be the inventor of one tenth of the doctrines of the *Lehre von den Augenkrankheiten*, but by long contemplation they had been so thoroughly amalgamated as to form but one harmonious whole, and therefore we are rarely startled by the clash of conflicting theories; but, instead of being informed of what Richter or Schmidt *thought* about a fact, we are generally told simply what the fact *is*. We feel, in short, in every sentence, that we are in the venerable presence of one who has seen 30,000 cases, and not only seen, but understood them, and who consequently more usually quotes his predecessors in order to correct their errors, than to defend himself by their authority. Of course we do not blame Mr. Lawrence for consulting the oracles of ophthalmic science, and looking at nature, as Dryden phrases it, "through the spectacles of books;" but we wish that he had done this only to improve his own art of seeing, and had taken off his glasses sooner. However, the treatise before us, though by no means of so high an order as Beer's extraordinary work, is compactly put together; and hence, with the exception of sundry addenda, whose unseemly presence at the end of the book might be compared to rough blocks left by a careless architect to deface the exterior of some splendid mansion, the work has a complete and finished air; and in this, as well as other respects, may be favourably contrasted with the crude notes which, under the name of books, are daily thrust upon the medical reader. Mr. Lawrence does not seem to have exactly made up his mind whether the diseases of the eye ought to form a separate branch of the healing art or not; he says,

"Although the importance of the subject must be admitted, it may be doubted whether the ophthalmic branch ought to be separated from the rest of medicine and surgery, as it must be, to a certain extent, by devoting to it separate courses of lectures and treatises, and by instituting ophthalmic hospitals. The diseases of the eye, in general hospitals, are inadequate, from the smallness of their number, to the purposes of practical study, particularly that of exemplifying the various operations. Thus these institutions have been inefficient in reference to this important department. As the general body of surgeons did not understand diseases of the eye, the public naturally resorted to oculists, who, seeing such cases in greater numbers, became better acquainted with the symptoms, diagnosis, and treatment; and especially more skilful in the operative department. At the same time, the subject being imperfectly understood, was neglected in the general surgical courses, in which many important affections were entirely unnoticed, and the whole very inadequately explained. Thus students, who resorted to London for the completion of their professional studies, had really no means of learning this important department of the profession, which was tacitly abandoned, even by the hospital surgeons, and turned over to the oculists. The latter not being conversant with the principles derived from anatomy, physiology, and general pathology, attended merely to the organ, and relied almost exclusively on what is comparatively of little importance,—local treatment. Hence ophthalmic surgery being in a manner dismembered from the general science, was reduced to a very low ebb. Until within a few years, it was in this country, at least, in a state of almost total darkness.

"It thus became desirable to establish an express and distinct school for ophthalmic surgery; not because the principles of treatment differ from those applicable to disease in general, nor because any peculiar mode of study is required, but in order to supply a deficiency in the existing sources of professional instruction; to provide, for the diseases of this important organ, those means of information which the general hospitals neither do, nor could provide, consistently with the requisite attention to their other important objects. This proceeding, which at first view seems calculated to complete and perpetuate the separation, was the only rational mode of re-uniting ophthalmic practice to general surgery." (P. 3.)

The oculists, it seems, became better operators than the hospital surgeons, and why? Because, although the latter were continually operating, it was not on the eye; and when they tried their hand on this small organ, it was found that their style was too coarse, too gross,—that they were deficient in minute dexterity. It does not appear, from Mr. Lawrence's statement, why the general hospitals could not provide means of information on ophthalmic subjects, if the principles of treatment do not "differ from those applicable to disease in general," and "no peculiar mode of study is required." The solution of this riddle, however, is not very difficult: surgery, like other arts, is improved by the division of labour; the minuteness of the subdivision increasing with the greatness of the surgeon's field of action, and it certainly is of advantage to the morbid public of London that their cataracts should be extracted or depressed by ten rather than by two hundred operators, especially if the ten are genuine surgeons, not *mere* oculists. Mr. Lawrence too is not quite correct in saying that ophthalmic surgery "*was reduced to a very low ebb.*" It had never been higher; and its state at the period to which Mr. L. refers, though low, was an improvement on its former one. Ophthalmic surgery was then in a state of transition; for the whole domain of medicine and surgery is so vast, that large tracts have always remained in a very uncultivated state, encouraging quacks and mountebanks to adopt the names of aurists, oculists, rupture-doctors, dentists, &c., as a means of preying on the commonwealth. With the progress of civilization and medical knowledge, the charlatan is gradually driven into unwilling decency, and even tries to pass himself off as a man of science, retaining, however, something of that mystery which is at once the essence and the badge of quackery. Ophthalmic surgery was in this state in London about thirty years since, when one of the principal oculists of the day did not blush to give prescriptions to his patients so written that they could be prepared only by one chemist. Such a man might be fairly considered as a *transitional* oculist, between the circumforaneous mountebank and the ophthalmic surgeon.

We next come to the history of the art, of which our author has given a very good account.

"*History.* The separation of the ophthalmic department from the rest of surgical practice has generally been considered of recent occurrence; it is, on the contrary, very ancient, and perhaps coëval with medicine itself. Among the Egyptians, to whom we trace the origin of arts and sciences, each class of diseases had its physician; and we find from Herodotus, that Cyrus sent to Amasis, the king of Egypt, for an oculist. The Greeks and the Romans had their oculists, as is evident, not only from their writings, but from the inscriptions on ancient marbles and seals. That Augustus and Tiberius were thus provided is apparent from the following inscriptions: *P. Attius Atimetus Augusti medicus ab oculis; Tit. Lyrius Tiberii medicus ocularius.** There is no doubt that oculists were at least as numerous in ancient Rome as in any modern city.

"The Greeks, the Romans, and the Arabians, were ignorant of anatomy, and could not therefore be acquainted with the essential nature of disease, that is, the altered structure of organs; nor connect with those changes, which really constitute disease, their appropriate external signs or symptoms. This disadvantage, however, is not so great in diseases of the eye as in many other affections, because most of them are externally visible, and obvious enough without anatomical knowledge. Hence the Greeks, who were good observers of nature, had noticed most forms of ophthalmic disease, in many instances described them well, and distinguished them accurately. The extent of their knowledge is evidenced by the imperishable records of language; for many of the diseases still bear the names given to them by the Greek writers. Celsus contains a summary of all that was known in his time. Although he was ignorant of the seat of cataract, he has described the operation of couching excellently and concisely, not omitting the important subjects of previous preparation and after treatment, for which his directions are judicious.

"In the fifteenth, sixteenth, seventeenth, and first half of the eighteenth century, the management of the diseases of the eye was left to quacks, to mountebanks, and itinerant practitioners. There were many of them, both in our own country and on the continent. It is not, however, worth while to draw their names and their writings from the oblivion to which they have been quietly consigned. The French writers on this subject, Maître-jan, St. Yves, and Janin, were more respectable than their contemporary brethren in other countries. The anatomy of the organ began to be more carefully cultivated by the Germans about the middle of the 18th century, when Zinn, Professor of Anatomy at Gottingen, published his excellent *Descriptio Anatomica Oculi Humani.* At a more recent period, Soemmerring produced his *Icones Oculi Humani*, a work of unrivalled beauty and accuracy, exhibiting an almost perfect set of engravings.

* These, and other similar inscriptions, are quoted by Haller from Gruter, and other authorities. Walch has collected every thing relating to the subject, in his *Sigillum Medici Ocularii Romani*; Jenæ, 1772. 8vo. Haller, *Bibl. Chir.* v. 1, lib. 1, § 24.

"The pathology of the eye was not much improved until within recent times. Boërhaave made an attempt on the subject, but his work *De Morbis Oculorum*, is very imperfect; indeed, some idea may be formed of the amount of his pathological knowledge, from his assertion, about mercury dissolving cataracts; he says, '*mercurius sæpe perfectas cataractas solvit.*' The Germans have had the greatest share in advancing our knowledge of ophthalmic diseases. Richter, Professor of Surgery at Göttingen, deservedly enjoyed the highest reputation in Germany, both for his general knowledge of the subject, and for his acquaintance with diseases of the eye, to which he paid great attention in his practice and writings. In his *Chirurgische Bibliothek* (*Bibliotheca Chirurgica*), which takes up the subject from the point where Haller's *Bibliotheca Chirurgica* leaves off, and comes dowu to 1797, he has carefully analysed all new publications on ophthalmic disease. The best account of the subject, at the time of its publication, is to be found in his *Anfangsgründe der Wundarzneykunst*, (*Elements of Surgery*,) of which the whole third volume and part of the second are devoted to diseases of the eye.

"But the most important era in the history of ophthalmic surgery, is the establishment of the Vienna school of ophthalmology. The Austrians have not only the honour of having instituted the first public establishment expressly appropriated to the advancement of this hitherto neglected branch of the profession, but of having preceded all the rest of Europe by many years. The views which directed the formation of this institution were so judicious, and the persons successively appointed to preside over it showed themselves so well fitted for the task, by their talents and knowledge, that the ophthalmic department of surgery has probably been more benefited by this school, than by the previous exertions of all other countries. The establishment owes its origin to Joseph Barth, a native of Malta, who repaired to Vienna in order to indulge a strong inclination, which he had felt from his earlier years, for the study of anatomy and surgery. His attention was accidentally directed to diseases of the eye, from seeing many persons in a state of hopeless blindness. His proficiency in this department was soon well known; and hence he was appointed lecturer on ophthalmic surgery in the university of Vienna, in 1773. Soon afterwards, certain wards were assigned for ophthalmic patients, in the general civil hospital, and a regular course of oral and clinical instruction was established. Barth wrote nothing except a short tract on the mode of performing extraction without an assistant; but he is considered to have set the example of those new and more correct views of ophthalmic disease, which are disclosed in the works of various German writers.

"Schmidt, who was educated by Barth, published a work on diseases of the lachrymal organs, and a valuable essay on Iritis. He also edited, in conjunction with Professor Himly, of Göttingen, an interesting periodical, devoted to ophthalmology (*Ophthalmolo-*

*gische Bibliothek*), of which three volumes appeared, from 1801 to 1807.

"Beer is more generally known than either Barth or Schmidt, as he was professor of ophthalmic medicine in the university of Vienna for many years, at a time when the high reputation of the school attracted students from all parts of Europe, and as he published many works. The last and principal of these, in 2 vols. 8vo., 1812 and 1817, devoted to the history, pathology, treatment, and operative surgery of the eye, was the most comprehensive work on the subject at the time of its publication. It contains accurate descriptions and histories, and consequently sound diagnostic precepts; but I cannot speak so favorably of the pathology and treatment. The compendium of Weller, which was translated by the late Dr. Monteath of Glasgow, is chiefly founded on the work of Beer." (P. 7.)

We are quite surprised that a man of Mr. Lawrence's reading should say that the Greeks, Romans, and Arabians were ignorant of anatomy; it is true that they knew less of it than the moderns: Celsus was not a Soemmering; but who would say that the Romans were ignorant of travelling, because the Iter ad Brundusium was not quite such a rapid affair as a journey to Exeter in the Quicksilver mail? Mr. Lawrence must have forgotten some of Celsus's descriptions; *e. g.* "Inde ima ventriculi pars paulum in dexteriorem partem conversa, in summum intestinum coarctatur. Hanc juncturam πυλωρὸν Græci vocant, quoniam portæ modo in inferiores partes ea, quæ excreturi sumus, emittit. Ab eâ jejunum intestinum incipit, non ita implicitum: cui tale vocabulum est, qui nunquam, quod accipit continet; sed protinus in inferiores partes transmittit. Inde tenuius intestinum est, in sinus vehementer implicitum: orbes vero ejus per membranulas singuli cum interioribus connectuntur, &c. Lib. iv. 1.

Nor is his osteology, in lib. viii. the work of a man ignorant of anatomy; but we will pass over this subject, though it is painful to us to find a man of Mr. Lawrence's reputation giving into the vulgar prejudices on this point.

Our author is well known to be a stanch supporter, or perhaps we should rather say, a violent partisan, of the antiphlogistic system, which is certainly set forth with sufficieut boldness in the following passage:

"A notion has prevailed that persons who live in London, or in other large towns, do not bear depletion well, and consequently that the loss of blood, which would be necessary in those who live in the country, would be improper in the inhabitants of the metropolis or extensive cities. I consider that this opinion is supported neither by experience nor argument. The inhabitants of London, from the highest to the lowest, with few exceptions, indulge their appetites freely; there are no small towns, nor any parts of the country, in which the consumption of animal food and stimulating liquors is more general. These habits, of which the injurious effects are aggravated in many instances by sedentary occupations or indolence, produce their natural consequences, namely, a plethoric state of the system, and an abundance of inflammatory disease, both of which circumstances will be immediately recognized, on attentive observation, whether among the higher or lower classes. I have not the least doubt that inflammations are as common and as violent among Londoners as among countrymen; and that they require the same treatment in both instances. The dread of depletion has been transmitted from one to another without examination or inquiry, and has led to an inert practice, under which disease has too often been suffered to proceed almost uncontrolled." (P. 107.)

It is hardly necessary to say that we entirely dissent from this theory; for it might be supposed even *à priori* that the inhabitants of the wretched courts and alleys of London, living on coarse food, drinking stimulating liquors, and continually exposed to what Dr. Ayre calls "mitigated famine," would be far below the bleeding point; and experience confirms this rational supposition. Instead of a dread of depletion having been transmitted from one to another without examination or inquiry, we have rather to complain that the passion for bleeding is still raging. Has not every hospital its Sangrado, save that harmless hot water has been replaced by disorganizing calomel? Nay, the strange monomania has infected the poor themselves; and nothing is more common than to hear some faint and flabby creature requesting to be bled or cupped, because he has a pain in his head, when he is suffering from the throbbing of debility, and cinchona, æther, and ammonia, are clearly indicated.

Mr. Lawrence has some judicious observations on the treatment of ophthalmia when the acute stage is passing into the chronic:

"When acute ophthalmia has been arrested by active treatment, it may be well to rest a little, and not to proceed immediately to new measures in order to guard against the imaginary evil of weakness. Allow an opportunity for the restorative powers of the part, and the constitution, to exert themselves. It is not necessary in medicine and surgery to be always doing something; to keep up an incessant fire of medicines and local applications. Nature will not stand still, even if the surgeon allows himself a little leisure; she proceeds, although the treatment be intermitted, in restoring the part to its healthy state.

"If the powers of the system should be really reduced by the long continuance of disease, and the necessary treatment, it may be expedient to adopt direct measures for invigorating the system. Nutritious diet, the moderate use of fermented liquors, good air and exercise, are the best restoratives: these should be combined with moderate use of the organ, which should be freely exposed to the air, and as much to the light as its irritability will allow. If the debility should seem to require, and more particularly if the patient should think that it calls for, the aid of the materia medica, we must employ the vegetable tonics and mineral acids." (P. 119.)

The method of treating the chronic form of the disease, that is to say, ninety-nine cases out of a hundred, by astringents, is given at some length, and is so important, that though our author is obviously incredulous, we shall make no apology for quoting the whole.

"*Use of stimuli and astringents.* The points which remain to be considered in the treatment of chronic ophthalmia are, the question as to the use of local stimulants and astringents; the time and circumstances under which, if useful, they are to be employed; and the particular remedies of this kind which are preferable. When the eye is preternaturally red, when it is weak and irritable, when exertion of it or exposure to light causes watering and pain, though it may be easy while at rest, stimulants and astringents are resorted to with the view of causing the distended vessels to contract, and thus removing what remains of inflammatory excitement. Of stimulants, the vinum opii, or vinous tincture of opium, has been much employed, both in this country and on the continent, in consequence of the recommendation of it by the late Mr. Ware; I do not know whether it was first introduced into practice by himself, or his partner, Mr. Wathen. The mode of employing it is to introduce half a drop, a drop, or two drops, between the palpebræ, so as to bring it into contact with the inflamed conjunctiva. The fluid may be taken up with a quill or a director, and, while the patient rests his head back, it may be dropped into the internal angle of the eye, so that when the lids are separated it may be diffused over the globe. The first effect is a sharp, smarting sensation, accompanied with a discharge of tears; but when this has gone off the patient generally feels relieved. The stimulus applied to the distended vessels is supposed to promote the contraction of them, and thus facilitate the recovery of their natural dimensions. It is employed once or twice a day. The vinum opii is the tinctura thebaica of the old London pharmacopœia, that is, of 1745, the ingredients of which were, an ounce of opium, half a drachm of cinnamon and of cloves, and half a pint of sherry wine. The opium and aromatics were macerated for eight days in the wine, and the tincture was then strained. In the modern pharmacopœias the tincture thebaica was omitted, and a spirituous tincture, the present tinctura opii, substituted for it. Mr. Ware ascribed a peculiar virtue to the combination of ingredients in the old preparation; he thought the spirituous tincture had not the same effect, and he found that opium alone, or wine alone, would not accomplish the purpose. I believe it was in consequence of Mr. Ware's recommendation, and the general use of the remedy in ophthalmia, that the College of Physicians again introduced vinum opii into their pharmacopœia; but it is singular that, as the efficacy of the remedy was so pointedly ascribed to the precise combination of ingredients in the old formula, it should have seemed fit to that learned body to diminish the quantity of opium one-half. Mr. Ware informs us, in a subsequent edition of his treatise, that this new form is just as efficacious as the old, in which opinion I quite agree with him. According to Mr. Ware's representations, it is a remedy of sovereign virtue. He seems to have used it indiscriminately in all cases of ophthalmia, both acute and chronic; in acute, combined with leeches, blistering, purging, and the treatment ordinarily called antiphlogistic. Without any particular specification of case, he directs the vinum opii to be dropped into the eye two or three times a day, in conjunction with other remedies. For my own part, I should never think of using it in acute ophthalmia; in such cases it would rather increase the inflammatory disturbance, though I must observe, at the same time, that it is not a very active remedy, and that it cannot do much mischief. Its employment should be restricted to cases of chronic inflammation, in which we have only to regret that its efficacy should fall so far short of the virtues ascribed to it. Having seen it often used, I hardly remember any case of a serious or obstinate kind, in which it alone has been decidedly effectual in arresting the disorder. It may afford a temporary relief to the patient, but I believe that if its use should be altogether abandoned, there would be no diminution either in the number of cures or in the time employed in effecting them.

"Weak brandy and water is a popular remedy for bad eyes, and is used without any discrimination of the nature or period of the affection. However, being applied externally, it is only to be considered as a cooling wash.

"The liquid laudanum of Sydenham is frequently mentioned by foreign writers; it is the old tinctura thebaica, with the addition of half an ounce of saffron.

"Various astringent metallic salts are employed in chronic ophthalmia, in the form of solution. Alum, in the proportion of from four to ten grains to an ounce of distilled water; sulphate of zinc or copper, from two to six or eight grains; nitrate of silver, one to six grains: oxymuriate of mercury, from one-eighth of a grain to one or two grains in the ounce of water. These solutions must be introduced between the palpebræ so as to come in contact with the inflamed surface. Their efficacy in common inflammation seems to be about equal to that of the vinum opii. In cases of purulent ophthalmia they have a more decided effect, as I shall have occasion to mention hereafter. The liquor plumbi subacetatis, undiluted, is used as an astringent. It might seem at first that it could not be safely applied to the eye in this state; but it is by no means an irritating application, though powerfully astringent. A French oculist, M. St. Ives, has proposed a remedy, which has been much employed on the continent under the name of lapis divinus. It is composed of a singular mixture of ingredients: an ounce of alum, nitre, and sulphate of copper, respectively, are fused together in a crucible: half a drachm of camphor is added towards the end of the process. A solution is made containing ten grains of the mixture in six ounces of water, the strength of which is to be increased according to circumstances. Such a mixture cannot of course have any effect differing from that of simple solutions of the metallic salts. A German writer, Conradi, has recommended a collyrium, which is often mentioned in the writings of his countrymen; it is composed of one grain of oxymuriate of mercury, six ounces of rose-water, one dram of mucilage of quince-seeds, and half a dram or a dram of the liquid laudanum of Sydenham.

"It may be observed generally, with respect to all these proposed remedies, that if active treatment be resorted to in the first instance, and followed up steadily, they are not wanted; and if insufficient means have been employed, so that a state of chronic inflammation is produced, this is a complaint which it is extremely difficult to remove, and which is not likely to yield to the vinum opii, or any remedies of that class.

"The use of strong astringents, more particularly the nitrate of silver, which has been found advantageous in inflammations of the conjunctiva, has been extended by Mr. Guthrie to other forms of ophthalmic inflammation, both acute and chronic. He proceeds on the principle 'of exciting an action greater, and of a different nature, to that already existing in the part.' He prefers the form of ointment to that of solution, on account of its more permanent action: and he has recommended the two following formulæ, viz.

"1. ℞. Argenti nitratis gr. ij. ad x.; liq. plumbi subacet. gtt. xv.: ung. cetacei ʒj.

"2. ℞. Hydrarg. oxymur. gr. iij. ad iv; liq. plumbi subacet. gtt. xv.; ung. cetacei. ʒj.

"The saline substances must be reduced to an impalpable powder, then mixed with the ointment on a slab, and the liquor plumbi added. It may be done in a glass mortar. These ointments are most stimulating when first made; they gradually become less so; but weeks elapse before they are inert.

"The manner of using either ointment is by introducing between the lids a portion, larger or smaller as the case may seem to require it, from the size of a large pin's head to that of a garden pea. The eye-lids being closed, are to be rubbed gently with the finger, so as to diffuse the dissolving ointment over the whole surface of the conjunctiva: a part of it usually, however, works out by the motion of the lids, and should be wiped off (if the nitrate of silver) to prevent its staining the skin. Both ointments cause pain:

in some persons it is considerable, in others less so, lasting from half an hour to an hour and a half; and, when the ointment is newly made, sometimes for four hours, and even until the next day. On the subsidence of the pain caused by the ointment, that which previously existed is found to be relieved, if not entirely removed; and on the subsequent day, the patient usually acknowledges the benefit he has received with regard to all the symptoms. When the application has been severe, and the patient very irritable, a state resembling white chemosis occasionally takes place, and appears formidable to a person unacquainted with the effect of the remedy: it soon, however, subsides. The eye should be fomented with warm anodyne fomentations. I rarely repeat the application until the third day: but the feelings of the patient are the best gnide, the return of some of the old sensations indicating the necessity for its use, which should be, if possible, anticipated. In some cases of acute inflammation, two or three applications will arrest the progress of a serious inflammation, and effect a cure. In chronic cases the ointment must be continued for a considerable time, and occasionally alternated with other remedies. Where it creates a state of regularly increased irritation, as it sometimes will do, cupping, purgatives, &c. are of service, when the remedies may be again resorted to.' Mr. Guthrie has generally used purgatives, but has sometimes found the ointments successful in serious complaints without any internal medicine. Sometimes they have disagreed altogether. In the *London Medical and Physical Journal*, New Series, No. 27, from which the preceding account is derived, as well as in the 31st No. of the same work, numerous cases are recited in illustration of the treatment. They are chiefly instances of chronic inflammation, purulent, common, and strumous, with thickening of the conjunctiva, opacity, vascularity, and ulcers of the cornea. At present, Mr. Guthrie seems to employ almost exclusively the ten-grain nitrate of silver ointment." (P. 120.)

Having ourselves witnessed the great success which attended the use of Mr. Guthrie's *Unguentum magicum* at the Eye Institution, we can strongly recommend this and similar remedies to our readers; for it is always satisfactory to have a stimulant which will render unnecessary the abstraction of blood from the wan and exhausted beings who come to a London dispensary.

The reason why the College of Physicians diminished by one half the quantity of opium in the vinum opii is very obvious. At present the vinum and the tincture differ but little in narcotic power, but had the former been twice the strength of the latter, an accidental substitution might have been dangerous, or even fatal.*

* Every one recollects the cases of the Parisian epileptics, who were poisoned by syrup of Prussic acid; a weak syrup having been intended, but a stronger one of the same name given.

Our author's abstract of the history and statistics of the Egyptian or purulent ophthalmia, is interesting.

"Purulent ophthalmia in subjects beyond the age of infancy is the same affection as that last described, only modified in its course, duration, and effects, by age; and requiring corresponding modifications in treatment. It is originally and essentially an affection of the mucous membrane of the eyelids; that is, inflammation with puriform discharge. It is often confined throughout to its original seat; more generally it extends to the conjunctiva oculi, when, if neglected or improperly treated, and sometimes, in spite of all the means that can be employed, it reaches the globe itself, producing in the cornea and iris injurious and destructive effects, similar to those which take place in newly-born children.

"When we consider its marked character and serious consequences, it seems strange that it should so long have escaped notice. Yet our knowledge of it is subsequent to that more extensive intercourse with Egypt which took place during the contest for its possession between France and this country. I know of no clear description of the complaint previous to this epocha. Scarpa does not mention it in his first edition, bearing date 1801, and has only a single paragraph on it, an additional one, in his last or 5th edition of 1818. Mr. Ware does not allude to it until long after the publications by the English army surgeons, subsequently to the evacuation of Egypt by our troops. Richter, who seems to have observed diseases of the eye with the greatest attention, for a long series of years, and who has described them with great fidelity, has not noticed this affection, which is not mentioned by Beer, nor by others of the Vienna school. Beer has entirely passed it over in his first edition of 1793: in the second edition of 1812-1816, he only alludes to it in a paragraph, in which he mentions that he had been long anxious to procure accurate information on the subject, and that his wishes had at last been gratified by a work of Assalini, which had convinced him that the complaint was merely inflammation of the glands of the eye-lids, (*blephar-ophthalmitis glandulosa*, that is, catarrhal inflammation of the lids,) rendered violent by the peculiar local circumstances, and passing quickly, in consequence of the unsuitable treatment of the natives, and of the French and English army surgeons, into blepharo-blennorrhœa, and ophthalmo-blennorhæa.

"The following circumstances will sufficiently prove the importance of the subject.

"Assalini states that two thirds of the French army were affected with the complaint at one time.

"Dr. Vetch informs us, in his interesting *Account of the Ophthalmia, which has appeared in England since the return of the British army from Egypt*, that 'the total strength of the second battalion of the 52d was somewhat above seven hundred men; six hundred and thirty-six cases of ophthalmia, including relapses, were admitted into the hospital, from August 1805, when the disease commenced, till the same month in 1806; of these fifty were dismissed with the loss of both eyes, and forty with that of one.'

"The ophthalmia depôt, under the care of this able physician, contained, in the summer of 1808, upwards of nine hundred cases from more than forty different corps.

"Cases of purulent ophthalmia had occurred in the 1st battalion of the 52d, when it went to Sicily, in 1806. It continued to suffer there. A part of the army of Sicily, which had been detached to Egypt, brought back with it fresh infection. From this station more than one hundred and thirty cases were sent home totally blind.

"It appears, from the returns of Chelsea and Kilmainham hospitals, that 2,317 soldiers were, on the 1st of December, 1810, a burthen upon the public from blindness, in consequence of ophthalmia. Those soldiers who have lost the sight of one eye are not included in the number above stated.

"In 1804, within nine months from April to December inclusive, nearly four hundred cases of purulent ophthalmia occurred at the Royal Military Asylum; and from that time to the end of 1810, upwards of nine hundred additional cases had taken place in the same establishment, without including relapses.

"Some years ago this alarming complaint broke out in a large boy's school in Yorkshire. Blindness of one or both eyes, or serious injury to sight, from corneal opacities or other causes, took place in nearly twenty instances. We cannot suppose that the proportion of unfavourable results would have been so considerable, if proper treatment had been adopted; for, in the Military Asylum, where the cases were so numerous, only six lost the sight of both eyes, and twelve the sight of one eye.

"Mueller treated 1,604 cases, including two hundred relapses, in the Prussian garrison of Mentz, in three years and a half; 1,344 were restored to the service perfectly well; fifteen became blind with both eyes, ten by staphyloma, three by entire suppuration of the cornea, one by leucoma, one by dropsy of the globe. Eighteen remained with impaired vision of both eyes; six by leucoma, four by cicatrix of the cornea with synechia anterior; six by suppuration of the cornea and opacities; one by pannus. Twenty-six remained blind of one eye; fifteen by staphyloma; one by cicatrix of the cornea, with synechia anterior; nine by opacities; one by pterygium." (P. 177.)

We shall not give any extracts from the account of gonorrhœal ophthalmia, as most of our readers are probably acquainted with Mr. Lawrence's work on that disease. Some of the patients, we recollect, lost 120 or 140 ounces of blood in a few days. It would be interesting to the medical philosopher to learn what became of their constitutions, and whether they really recovered in the true sense of the word.

In treating iritis, both in its acute and chronic forms, Mr. Lawrence relies on mercury, and has never tried turpentine.

The science of medical statistics is so entirely in its infancy, that it would be in vain to ask whether the cases we are about to quote exhibit an unusual result, and whether, in the treatment of iritis, we should follow Beer or Lawrence?

"CASE. A lady, of twenty-five, tall, with light hair and irides, who had been in the habit of spending much time in needle-work, had experienced, for some months before she consulted me, dimness of sight in the right eye, which had come on gradually, without pain or redness, except that the eye had been a little bloodshot for a day or two in the very beginning, after which the appearance went off. For the previous six weeks she had been judiciously treated by a physician: venesection, leeches to the temple, aperients, and mercury carried to the length of salivation, had been employed. There were three adhesions of the right pupil, scarcely discernible in its natural state, but rendered obvious by the use of belladonna: all useful vision was lost in this eye. Two adhesions existed in the left eye, and vision was dim. No pain or redness had ever been noticed in this eye. The irides, pupils, and all visible parts of both eyes, were perfectly natural in all other respects.

"CASE. A young lady, of delicate frame and constitution, of great information and accomplishments, who habitually devoted a large portion of her time to music, reading, drawing, and fine needle-work, found, on looking at a picture with the left eye shut, that she had lost the sight of the right. She had experienced no uneasiness in it; there had been no redness, nor any other change to attract the notice of her friends. A gentleman since dead, who had great reputation in this department of practice, was consulted. He said that the eye had probably been originally defective; and, in answer to an inquiry on that subject, he observed that there was no necessity for restriction in the use of the other eye. As this opinion, which accorded with the inclination of the patient, was acted upon, the left eye soon became diseased, and the case was placed under my care. I found inflammation of the left iris, with adhesion of the pupil, slight external redness, and some pain. In the right eye, the iris was slightly altered in appearance, and the pupil fringed with slender dark adhesions; the aperture itself was clear, but vision was extinct. A mild antiphlogistic treatment, followed by the use of mercury, restored the sight of the left eye; it was necessary to continue the mercurial course for some weeks, although the patient's friends had in the first instance entertained great apprehensions of the remedy, on account of her delicate constitution and supposed consumptive tendency. A relapse took place at the end of the year, when the employment of mercury was again successful, but not till after it had been used for many weeks. In a third relapse, the patient again used mercury, not so much from the recommendation of her medical advisers, as from her own conviction that it was the only means of restoring her sight. She employed it by friction, and persevered for five months before vision was restored. These unusually long and repeated mercurial courses produced none of the anticipated injurious effects on health. Disease returned again, and at last destroyed all useful vision.

"Extension of inflammation to the posterior tunics is most to be feared in acute iritis; but that the chronic form of the disease is not exempt from this danger, is rendered evident by the two foregoing histories." (P. 315.)

Again, speaking of syphilitic iritis, our author says,

"It is but rarely seen as a symptom of syphilis in infants: numerous children labouring under this disease have come under my observation, but iritis has occurred in two instances only. In one of them there were excoriations and ulcerations round the anus. The iris had lost its brilliancy, and become dark-coloured; the pupil was slightly contracted, and there was some redness of the sclerotica. On the other case I was consulted by letter from the country. The father had had primary venereal sores before marriage. In a few weeks after birth, the child had an eruption all over the body, wasted, and seemed on the point of dying. It got well under the use of mercury in very small quantities. In a few weeks more, severe inflammation of the eyes came on; mercury was employed in the same manner; the inflammation was arrested, but the child remained blind. I saw it some weeks after. Both pupils were fixed, and moderately contracted. An opaque body, which was not a cataract, was seen behind one; the other was clear. Both eyes were blind." (P. 317.)

In one instance, "syphilitic iritis, or rather syphilitic inflammation of the internal tunics, occurred as a secondary symptom, in conjunction with scaly eruption, after the infection of a chap on the hand by the contact of discharge from a sore in delivery." (P. 317.)

Under the name of retinitis, Mr. Lawrence gives several cases of amaurosis cured or relieved by depletion. Rosas says that the affection does not usually extend over the whole retina, but that it is found, on examination after death, to be confined to the neighbourhood of the yellow spot. Mr. L. observes, on the other hand, that we are not acquainted with the phenomena of retinitis so well as with those of iritis, because the part affected is out of sight, and the disease does not terminate fatally. The diagnosis he gives is not very satisfactory: "Pain and impaired vision are its leading circumstances; the pupil is at first contracted, then enlarged." (P. 323.)

Mr. Lawrence considers the subject of amaurosis under three divisions, according as it depends on affection of the sensorium, the optic nerve, or the retina. The following cases belong to the first division:

"CASE I. *Imperfect amaurosis remaining after violent disorder of the head.* In August, 1826, I saw a lady, forty-two years of age, who was still menstruating. Twelve or fourteen months previously she had been reduced to so dangerous a state by violent disorder of the head, that her medical attendant had discontinued his visits, stating that further efforts were useless, and death inevitable. The gentleman who came with her to my house, and who had been called in on this occasion, found the patient comatous, and discharging her urine and feces unconsciously. By leeches to the head, and other antiphlogistic treatment, her state was improved, and she ultimately recovered, but with loss of sight in both eyes. I found the pupils of middle size, with scarcely any sensible change or variations in the quantity of light. The right was a little altered in figure, and slightly dull in colour. She could make out, one after the other, capital letters of the second size in the title-page of an octavo book. Her health was excellent. Before the illness, she had suffered much for years from headach; since her recovery she had been thrown from a gig, and freely bled and evacuated. This depletion had completely removed the pain in the head.

CASE II. *Complete amaurosis, with perfect motion of the irides occurring with violent pains of the head.* A girl of eight was brought to me by her mother totally blind. The sight had been gradually lost, with violent pains in the head, three years previously, when she had not begun to menstruate. She had been treated at the time by cupping and other corresponding measures. The pupils were rather dilated, but the appearance of the eyes was in other respects perfectly healthy; and the irides acted well. She menstruated regularly, but had still pains in the head. The latter were removed by a course of the hydrargyrus cum creta with aperients; but vision was not improved.

"CASE III. *Complete amaurosis produced suddenly, by sensorial congestion.* A patient in St. Bartholomew's Hospital, about thirty years of age, with enlargement of the testicle, had been directed to rub a little mercurial liniment on the part daily, and had done this four or five times, when salivation occurred. He felt indisposed in the evening of Saturday, but went to bed without making any complaint. He awoke in the middle of the night with great pain in the head, and feeling very ill. He got up, and thought that the candle, usually kept burning during the night, had gone out, for he could not see it; in fact, his sight, which had been perfect when he went to bed, was lost. The house surgeon found him with a full, strong, and frequent pulse, and bled him. He afterwards administered an emetic, which was acting when I saw him at twelve o'clock, Sunday. The pulse was still full and strong, and there was great pain in the head. The pupils were about the middle state, the irides nearly but not quite motionless, and vision so completely extinct, that when a lighted candle was held near the eyes, the patient was not sensible of its presence. I ordered repetition of bleeding, and the application of a large blister at the nape. These means were again repeated. In a week vision was restored, and in a fortnight the patient left the hospital quite well." (P. 493.)

In the third section our author gives a number of cases to illustrate his principle of treatment. We will content ourselves with quoting the second one. It shows the advantage of the antiphlogistic system of treatment in a plethoric subject, and is also a favourable specimen of our author's pithy manner of narrating a case.

Case II. *Imperfect amaurosis with plethora, before menstruation had begun.* Another young woman came to the infirmary with impaired vision; her countenance was red and flushed, and she complained of considerable pain in the head; there was evident congestion about this part. She was about fifteen years of age; menstruation had not commenced, and the consequence was a plethoric condition of the system. She was bled two or three times, purged actively, and continued this plan of treatment for two or three weeks before the retina was relieved, when the menses appeared, and she completely recovered. (P. 532.)

These copious extracts will be sufficient to show the style of Mr. Lawrence's work, which, in conclusion, we recommend to our readers as a most valuable repertory of ophthalmic practice.

